# Geospatial disparities in pediatric heart failure care in China: a multicenter cohort study

**DOI:** 10.3389/fpubh.2025.1737404

**Published:** 2026-01-16

**Authors:** Muhammad Junaid Akram, Yin Yue, Asad Nawaz, Zahoor Elahi, Wenjing Yuan, Lingjuan Liu, Bo Pan, Yuxing Yuan, Jie Tian

**Affiliations:** 1 Department of Cardiology, Children’s Hospital of Chongqing Medical University, National Clinical Research Center for Children and Adolescents’ Health and Diseases, Ministry of Education Key Laboratory of Child Development and Disorders, International Science and Technology Cooperation Base of Child Development and Critical Disorders, Chongqing, China; 2 Key Laboratory of Children's Important Organ Development and Diseases of Chongqing Municipal Health Commission, National Clinical Key Cardiovascular Specialty, Chongqing, China

**Keywords:** geospatial disparities, guideline-directed medical therapy, healthcare inequities, length of stay, mortality, pediatric heart failure

## Abstract

**Background:**

Pediatric heart failure (PHF) is a lethal syndrome with a distinct pathophysiology from adult heart failure, posing a significant public health challenge in China. However, the impact of the nation’s profound geospatial healthcare disparities on this vulnerable population remains unquantified. This national multicenter cohort study aimed to systematically examine the association between geographic location and PHF patient profiles, management, and survival outcomes.

**Methods:**

We conducted a retrospective analysis of 2,903 pediatric inpatients (≤18 years) with a primary HF diagnosis from 30 centers (2013–2022). Patients with >20% missing data were excluded. Geospatial analysis stratified cohorts into Eastern, Western, and Central China. The primary outcome was in-hospital mortality, analyzed using multivariable logistic regression adjusted for key clinical confounders including age, etiology, and disease severity. Length of stay (LOS) was analyzed using a Gamma generalized linear model. All analyses were performed using Python (version 3.12).

**Results:**

Profound geospatial disparities were identified. Western patients presented significantly younger (median 6.93 months) with a higher prevalence of complex congenital heart disease (39.2%) and more severe clinical status (85.9% ROSS Class III-IV). In contrast, cardiomyopathy was the dominant etiology in the East (42.2%). Utilization of guideline-directed medical therapy (GDMT) was lowest in non-Eastern regions. Critically, geographic region was independently associated with mortality. Compared to the East, the adjusted odds of death were 2.58-fold higher in the West (95% CI: 1.5–4.45) and 3.54-fold higher in the Central region (95% CI: 2.11–5.94).

**Conclusion:**

This study provides robust, national-level evidence that geographic location in China is a potent independent predictor of survival for children with heart failure, revealing a tiered healthcare landscape. These findings underscore an urgent public health imperative for targeted interventions to mitigate these disparities and establish equitable care systems through regional capacity building and standardized referral pathways.

## 1 Introduction

Pediatric heart failure (PHF) is a life-threatening clinical syndrome and a leading cause of mortality in children, representing a final common pathway for diverse etiologies including congenital heart disease (CHD), cardiomyopathies, and myocarditis (1–4). Unlike adult heart failure, PHF pathophysiology is rooted in developmental and genetic aberrations, often leading to aggressive clinical courses (5, 6). The global incidence of PHF is estimated at 0.87–7.4 per 100,000 children. In China, with a CHD prevalence approaching 9 per 1,000 live births, the absolute disease burden is immense, creating a critical public health challenge (7–9).

The management of this high-risk population is contingent upon a centralized, specialized care model. Optimal outcomes require timely access to tertiary pediatric cardiac centers capable of providing advanced diagnostics, pharmacotherapy, and interventions, including mechanical circulatory support and transplantation (10, 11). However, China’s vast geography and uneven socioeconomic development have resulted in profound geospatial disparities in healthcare resource distribution. Robust evidence confirms that children in rural and less developed regions face significant barriers to care, including diagnostic delays, financial constraints, and limited access to specialized cardiac expertise, leading to inequitable health outcomes for a range of pediatric conditions (12–16).

While these systemic vulnerabilities are recognized, the magnitude and clinical consequences of these disparities specifically for pediatric heart failure across China have not been comprehensively quantified at a broader level. A detailed understanding of how geographic location influences patient characteristics, disease severity, treatment patterns, and ultimate survival is a critical first step toward addressing these inequities. Therefore, this study aims to conduct a national geospatial analysis of PHF care in China. By mapping the variations in clinical presentation, management, and outcomes across different regions, this investigation seeks to identify critical gaps in the care continuum and inform the development of a more equitable system for this vulnerable population.

## 2 Methodology

### 2.1 Study design and setting

We conducted a multicenter, retrospective cohort analysis to evaluate geospatial disparities in pediatric heart failure care across China. This investigation, supported by the National Center for Children’s Health Clinical Research, involved 30 medical centers from 20 Chinese provinces, ensuring broad geographic and socioeconomic representation. Hospitals were categorized into three major geographic regions (East, West, Central) based on the National Bureau of Statistics classification of China’s economic zones. A full list of participating centers and their assigned regions is provided in Supplementary Table S2. The study period encompassed 10 years, from January 2013 to December 2022, facilitating a comparative assessment of healthcare delivery across regions. Eligible patients were included upon their index admission at any point during this 10-year window, with follow-up for each patient spanning from admission until hospital discharge or in-hospital death.

### 2.2 Ethical considerations

The study protocol was approved by the Institutional Review Board of Chongqing Medical University (Approval No. 2020.160). A waiver for informed consent was granted due to the retrospective design and use of de-identified data. All procedures adhered to the ethical principles of the Declaration of Helsinki and national data security statutes. Patient identifiers were removed prior to central database compilation to safeguard confidentiality.

### 2.3 Participant selection

**Figure 1:**
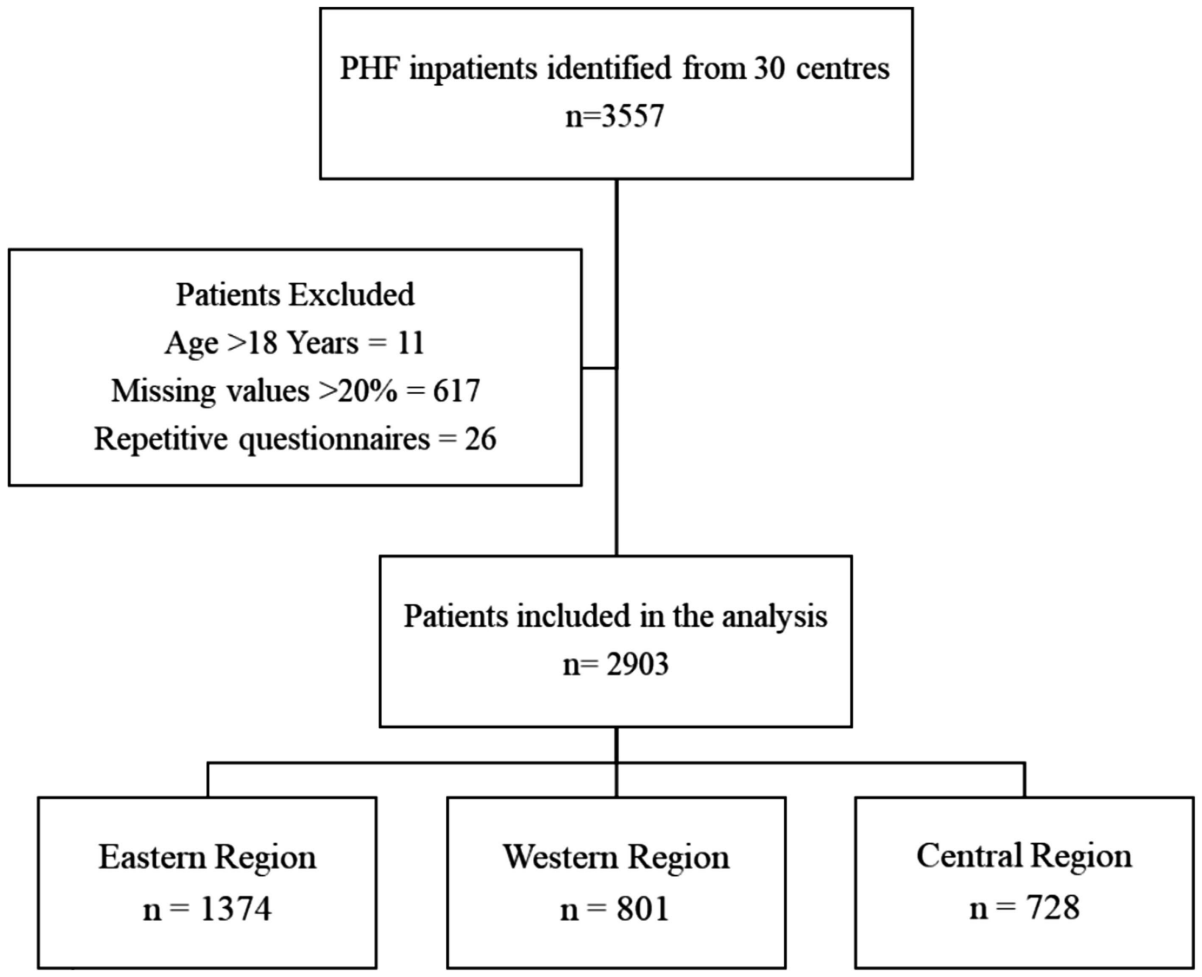
Study cohort derivation.

The study population comprised pediatric inpatients (≤18 years) with a primary diagnosis of heart failure, established according to national Chinese guidelines. To ensure data quality and analytical validity, exclusion criteria were rigorously applied. These included: (1) medical records with substantial missing data (≥20%), (2) duplicate patient entries, (3) individuals aged over 18 years, and (4) records with chronologically inconsistent admission or discharge data. The application of these criteria yielded a final analytical cohort of 2,903 patients, with the selection process detailed in a consort-style flowchart (Figure 1).

Flowchart illustrating the participant selection process for the multicenter, retrospective study on geospatial disparities in pediatric heart failure care. The application of pre-specified exclusion criteria to the initial population (*n* = 3,557) resulted in the final analytical cohort (*n* = 2,903). The primary (in-hospital mortality) and secondary (LOS) outcomes for this cohort were analyzed using multivariable regression models adjusted for clinical confounders to evaluate regional differences.

### 2.4 Data acquisition, management and processing

A standardized data collection protocol, derived from our previously established and validated methodology, was implemented across all centers to ensure uniformity in data acquisition and handling (6). Clinical and demographic variables, captured at the point of admission, were abstracted from the Hospital Information Systems (HIS) of participating institutions. The comorbidity ‘severe infection’ was defined by the presence of a physician diagnosis of severe pneumonia, sepsis, bacteremia, or septicemia. The primary outcome, in-hospital mortality, was ascertained from the HIS discharge status. LOS, defined as the number of days from admission to discharge, was collected as a key secondary outcome measure. A standardized, centralized Microsoft Access database (v16) facilitated uniform data collection. To ensure fidelity, a two-stage verification process was implemented, involving trained data abstractors who performed blinded double-entry for a subset of records. A structured, multi-step protocol was employed to manage incomplete data. Initially, clinical variables with a high degree of missingness (≥70%) were excluded from the dataset; these included E velocity, A velocity, E/A ratio, HDL, LDL, IVRT, and cTnT. Subsequently, individual patient records with >20% missing data were removed from the cohort. For the remaining minor missing values in the final dataset, Multiple Imputation by Chained Equations (MICE) was applied for conservative imputation.

### 2.5 Statistical analysis

All analyses were executed using Python (v3.12) with standard scientific libraries (Pandas, NumPy, SciPy, StatsModels). Continuous variables, predominantly non-normally distributed, were compared using the Kruskal-Wallis H-test for multi-group analyses, with post-hoc pairwise Mann–Whitney U tests adjusted by the Bonferroni method. Categorical variables were compared using the Chi-square test, with similar post-hoc pairwise testing. Multivariable regression models were constructed to evaluate the independent association of geographic region with the primary and secondary outcomes. Mortality was analyzed using multivariable logistic regression, with results presented as adjusted odds ratios (aOR) and 95% confidence intervals (CI). LOS was analyzed using multivariable generalized linear models (GLM) with gamma distribution and log link function, selected after distributional analysis confirmed severe right-skewness (skewness = 3.411, Shapiro–Wilk *p* < 0.001). All models were adjusted for pre-specified potential confounders, including age group, sex, CHD, cardiomyopathy, left ventricular ejection fraction (LVEF), ROSS classification, and log-transformed B-type Natriuretic Peptide (log_BNP). A two-sided *p*-value of < 0.05 defined statistical significance for all tests.

## 3 Results

### 3.1 Demographics and clinical presentation

The final analytic cohort comprised 2,903 pediatric patients with heart failure. Profound geospatial disparities were observed in baseline characteristics across the Eastern, Western, and Central regions of China (Table 1). The patient population in the Western region was significantly younger (median age: 6.93 months) compared to the East (21.83 months) and Central (12.97 months) regions, with a higher proportion of newborns (*p* < 0.001). Anthropometric measurements at admission, including weight and height, were consistently lowest in the West (*p* < 0.001).

**Table 1:** Patient characteristics and clinical profiles by geographic region.

Variable	Overall (*N* = 2,903) *n* (%)	East (*n* = 1,374) *n* (%)	West (*n* = 801) *n* (%)	Central (*n* = 728) *n* (%)	*p*-value
Demographic features					
Gender (Male)	1,501 (51.7)	721 (52.5)	389 (48.6)	391 (53.7)	0.097
Family history of heart disease	258 (8.9)	157 (11.4)*	42 (5.2)	59 (8.1)	<0.001
History of cardiovascular surgery	232 (8.0)	146 (10.6)*	32 (4.0)‡	54 (7.4)	<0.001
BMI					
BMI normal	2,237 (77.1)	1,077 (78.4)	594 (74.2)	566 (77.7)	0.068
BMI underweight	493 (17.0)	227 (16.5)	163 (20.3)‡	103 (14.1)	0.005
BMI obese	173 (6.0)	70 (5.1)†	44 (5.5)	59 (8.1)	0.017
Age Group					
Newborn	212 (7.3)	45 (3.3)*†	107 (13.4)‡	60 (8.2)	<0.001
Infant	1,618 (55.7)	728 (53.0)*	499 (62.3)‡	391 (53.7)	<0.001
Child	766 (26.4)	436 (31.7)*	131 (16.4)‡	199 (27.3)	<0.001
Adolescents	307 (10.6)	165 (12.0)*	64 (8.0)	78 (10.7)	0.013
Clinical features					
ROSS III-IV	2,120 (73.0)	940 (68.4)*	688 (85.9)‡	492 (67.6)	<0.001
Respiratory symptoms	2,185 (75.3)	931 (67.8)*†	688 (85.9)‡	566 (77.7)	<0.001
Gastrointestinal symptoms	717 (24.7)	346 (25.2)	190 (23.7)	181 (24.9)	0.743
Systemic venous congestion	2,153 (74.2)	978 (71.2)*	675 (84.3)‡	500 (68.7)	<0.001
Interrupted feeding	816 (28.1)	348 (25.3)	252 (31.5)	216 (29.7)	0.005
Pallor	867 (29.9)	380 (27.7)†	225 (28.1)‡	262 (36.0)	<0.001
Restlessness	613 (21.1)	216 (15.7)*†	208 (26.0)	189 (26.0)	<0.001
Growth retardation	1,144 (39.4)	483 (35.2)*	412 (51.4)‡	249 (34.2)	<0.001
Severe infection	850 (29.3)	264 (19.2)*†	235 (29.3)‡	351 (48.2)	<0.001
Heart failure types and etiologies					
Chronic heart failure	1,029 (35.4)	559 (40.7)†	350 (43.7)‡	120 (16.5)	<0.001
CHD	1,062 (36.6)	420 (30.6)*	453 (56.6) ‡	189 (26.0)	<0.001
Simple CHD	331 (11.4)	126 (9.2)*	139 (17.4)‡	66 (9.1)	<0.001
Complex CHD	731 (25.2)	294 (21.4)*†	314 (39.2)‡	123 (16.9)	<0.001
VSD	427 (14.7)	135 (9.8)*	219 (27.3)‡	73 (10.0)	<0.001
PDA	297 (10.2)	85 (6.2)*†	144 (18.0)‡	68 (9.3)	<0.001
Valvular malformations	159 (5.5)	95 (6.9)†	49 (6.1)‡	15 (2.1)	<0.001
Cardiomyopathy	978 (33.7)	580 (42.2)*	121 (15.1)‡	277 (38.0)	<0.001
HCM	62 (2.1)	34 (2.5)	11 (1.4)	17 (2.3)	0.21
DCM	461 (15.9)	268 (19.5)*	49 (6.1)‡	144 (19.8)	<0.001
RCM	47 (1.6)	34 (2.5)†	5 (0.6)	8 (1.1)	0.002
ARVC	49 (1.7)	34 (2.5)	8 (1.0)	7 (1.0)	0.008
Myocardial densification insufficiency	207 (7.1)	121 (8.8)*	24 (3.0)‡	62 (8.5)	<0.001
Endocardial elasto-fibrillar hyperplasia	155 (5.3)	84 (6.1)*	22 (2.7)‡	49 (6.7)	<0.001
Myocarditis	184 (6.3)	84 (6.1)	48 (6.0)	52 (7.1)	0.585
Cardiomegaly	2,193 (75.5)	1,120 (81.5)*†	600 (74.9)‡	473 (65.0)	<0.001
Pulmonary congestion	853 (29.4)	421 (30.6)	225 (28.1)	207 (28.4)	0.366
Pulmonary hypoperfusion	14 (0.5)	7 (0.5)	1 (0.1)	6 (0.8)	0.14
Prominent aortic node	9 (0.3)	3 (0.2)	4 (0.5)	2 (0.3)	0.514
Prominent pulmonary artery segment	73 (2.5)	34 (2.5)	25 (3.1)	14 (1.9)	0.325
Supraventricular tachycardia	281 (9.7)	161 (11.7)*†	57 (7.1)	63 (8.7)	0.001
Ventricular tachycardia	169 (5.8)	81 (5.9)	40 (5.0)	48 (6.6)	0.405
Malignant arrhythmias	167 (5.8)	110 (8.0)*†	23 (2.9)	34 (4.7)	<0.001
Surgical interventions					
Moderate/severe valvular regurgitation	878 (30.2)	394 (28.7)*	299 (37.3)‡	185 (25.4)	<0.001
Occlusion/balloon dilatation	50 (1.7)	17 (1.2)*	23 (2.9)	10 (1.4)	0.013
Simple congenital heart thoracotomy	328 (11.3)	132 (9.6)*	120 (15.0)‡	76 (10.4)	<0.001
Radiofrequency ablation	32 (1.1)	25 (1.8)*	1 (0.1)	6 (0.8)	<0.001
Pacemaker	125 (4.3)	97 (7.1)*†	11 (1.4)	17 (2.3)	<0.001

Symbols indicate a statistically significant difference (*p* < 0.05) in a *post-hoc* pairwise comparison:

*Significant difference between East and West regions.

†Significant difference between East and Central regions.

‡Significant difference between West and Central regions.

BMI, Body Mass Index; VSD, Ventricular Septal Defect; PDA, Patent Ductus Arteriosus; HCM, Hypertrophic Cardiomyopathy; DCM, Dilated Cardiomyopathy; RCM, Restrictive Cardiomyopathy; ARVC, Arrhythmogenic Right Ventricular Cardiomyopathy.

The etiological profile of heart failure exhibited striking regional variation. CHD was the dominant etiology in the West, with significantly higher rates of complex CHD (39.2%), ventricular septal defects (VSD, 27.3%), and patent ductus arteriosus (PDA, 18.0%) compared to other regions (*p* < 0.001 for all). In contrast, cardiomyopathy was the leading etiology in the East (42.2%) and Central (38.0%) regions, versus only 15.1% in the West (*p* < 0.001). This was driven by higher rates of dilated cardiomyopathy (DCM) in the East and Central regions.

Disease severity and clinical presentation also differed markedly. The Western region demonstrated the highest acuity, with the greatest prevalence of ROSS Class III-IV heart failure (85.9%), respiratory symptoms (85.9%), and systemic venous congestion (84.3%) (*p* < 0.001). The Western region had the highest prevalence of chronic heart failure (43.7%), while the Central region had the lowest (16.5%). Comorbid severe infection was most common in the Central region (48.2%, *p* < 0.001).

### 3.2 Echocardiographic, hemodynamic, and biomarker profiles

Echocardiographic assessment revealed distinct pathophysiological phenotypes (Table 2). Consistent with their younger age and higher CHD burden, Western patients had smaller cardiac chamber dimensions (LA, RA, RV, LVDd, LVDs) but preserved systolic function, evidenced by higher median left ventricular ejection fraction (LVEF: 64%) and fractional shortening (LVFS: 33%) (*p* < 0.001 vs. other regions). Patients in the East had the most impaired systolic function (median LVEF: 46%). Pulmonary artery pressure was significantly elevated in the West (median PAP: 47.70 mmHg) compared to the East and Central regions (*p* < 0.001).

**Table 2:** Clinical and echocardiographic parameters by geographic region.

Variable	Overall median (IQR)	East median (IQR)	West median (IQR)	Central median (IQR)	*p*-value
Clinical features					
Age (Months)	12.83 (3.42–82.68)	21.83 (4.73–100.02)*†	6.93 (2.27–33.20)‡	12.97 (3.32–87.07)	<0.001
SBP (mmhg)	90.75 (83.00–102.00)	93.00 (86.00–104.00)*†	89.25 (81.00–97.00)‡	90.00 (80.00–102.00)	<0.001
DBP (mmhg)	55.00 (49.00–64.00)	56.79 (50.00–65.00)*	52.69 (46.00–60.00)‡	56.00 (49.00–65.00)	<0.001
HR (bmp)	135.00 (114.98–167.00)	130.00 (108.25–160.00)*†	139.00 (122.00–165.00)	140.00 (120.00–180.00)	<0.001
Echocardiographic parameters					
LA (mm)	19.78 (16.00–25.00)	20.00 (17.00–27.00)*†	18.00 (14.00–22.03)‡	20.00 (16.00–25.00)	<0.001
RA (mm)	23.00 (18.35–30.57)	25.00 (20.00–33.00)*†	20.00 (16.81–26.10)‡	22.00 (18.00–30.00)	<0.001
RV (mm)	15.67 (12.00–20.12)	16.25 (12.00–21.13)*†	14.00 (11.00–18.00)	14.62 (11.64–20.03)	<0.001
LVDd (mm)	33.15 (25.00–43.94)	37.00 (28.00–46.00)*†	29.00 (22.00–37.00)‡	32.00 (23.00–44.00)	<0.001
LVDs (mm)	24.00 (16.82–34.95)	29.00 (19.79–38.00)*†	19.00 (14.00–26.00)‡	22.63 (14.00–34.84)	<0.001
AO (mm)	13.00 (11.00–18.00)	14.00 (11.00–18.00)*†	13.00 (11.00–17.00)	12.32 (10.00–18.00)	<0.001
IVDd (mm)	5.90 (4.16–7.73)	6.00 (4.53–8.00)*†	5.20 (4.02–7.00)‡	6.00 (4.00–7.54)	<0.001
LVPWD (mm)	5.00 (4.00–6.00)	5.00 (4.00–6.31)*†	4.24 (3.87–5.46)‡	5.00 (4.00–6.00)	<0.001
LVEF (%)	54.00 (35.00–67.00)	46.00 (32.00–63.00)*†	64.00 (52.44–71.00)‡	52.00 (33.00–67.00)	<0.001
LVFS (%)	27.00 (18.00–36.00)	23.00 (16.00–33.00)*†	33.00 (26.58–39.00)‡	26.00 (16.00–36.00)	<0.001
PAP (mmhg)	44.24 (35.00–53.61)	43.64 (35.00–52.92)*†	47.70 (38.20–56.07)‡	42.00 (31.60–51.22)	<0.001

Data are presented as Median (IQR). Symbols indicate a statistically significant difference (*p* < 0.05) in a *post-hoc* pairwise comparison:

*Significant difference between East and West regions.

†Significant difference between East and Central regions.

‡Significant difference between West and Central regions.

SBP, Systolic Blood Pressure; DBP, Diastolic Blood Pressure; HR, Heart Rate; LA, Left Atrium; RA, Right Atrium; RV, Right Ventricle; LVDd, Left Ventricular Diastolic Diameter; LVDs, Left Ventricular Systolic Diameter; AO, Aorta; IVDd, Interventricular Diastolic Diameter; LVPWD, Left Ventricular Posterior Wall Diastolic Diameter; LVEF, Left Ventricular Ejection Fraction; LVFS, Left Ventricular Fractional Shortening; PAP, Pulmonary Artery Pressure.

Biomarker analysis further confirmed divergent clinical states (Table 3). B-type Natriuretic Peptide (BNP) levels were highest in the Central region (median: 2032.09 pg/mL) and lowest in the West (median: 775.30 pg/mL) (*p* < 0.001). This pattern of lower absolute BNP levels in the West, despite the highest clinical acuity scores, may reflect the distinct pathophysiology of volume-overload heart failure prevalent in this cohort. Myocardial injury, as measured by Creatine Kinase-MB (CK-MB), was also most pronounced in the Central region compared to the East and West (*p* < 0.001). Hepatic congestion or injury was more evident in the West and Central regions, with significantly higher alanine aminotransferase (ALT) levels (*p* < 0.001).

**Table 3:** Distinct biomarker profiles by geographic region.

Variable	Overall median (IQR)	East median (IQR)	West median (IQR)	Central median (IQR)	*p*-value
Cardiac biomarkers					
BNP, pg/ml	1,397 (324.1–3439.6)	1513.5 (440.5–3456.5)*†	775.30 (132.00–2,189)‡	2032.09 (501.85–5,000)	<0.001
NT-ProBNP, pg/ml	7692.4 (3421.2–16314.5)	7486.2 (2726.8–16169.7)†	7705.6 (4850.4–12,422)	7904.6 (2257.5–20957.7)	0.025
CK-MB, ug/L	8.00 (2.90–25.00)	8.90 (3.10–26.00)*†	4.22 (2.11–10.50)‡	16.43 (5.12–31.54)	<0.001
cTnI, ug/L	0.06 (0.01–2.51)	0.06 (0.01–1.15)	0.06 (0.01–1.27)	0.05 (0.00–40.08)	0.862
Liver & kidney function					
ALT, U/L	26.00 (17.70–46.50)	23.00 (16.00–39.00)*†	29.00 (19.10–51.90)	30.00 (18.27–52.70)	<0.001
AST, U/L	43.70 (31.55–66.00)	41.00 (30.58–60.00)*	49.10 (34.60–78.50)‡	42.30 (31.00–66.03)	<0.001
ALB, g/L	39.40 (34.90–43.20)	40.00 (36.30–43.50)*†	39.00 (33.50–43.70)	38.20 (33.30–42.10)	<0.001
ALP, U/L	191.00 (136.71–258.00)	191.00 (135.61–259.72)	190.50 (137.10–263.00)	193.00 (136.78–252.22)	0.865
Cr, umol/L	30.30 (23.00–45.00)	31.30 (23.66–44.50)*	28.10 (22.00–42.00)‡	31.55 (23.00–48.17)	<0.001
BUN, g/dL	4.61 (3.06–6.70)	5.00 (3.21–7.25)*†	4.25 (2.89–5.90)	4.50 (3.00–6.45)	<0.001
UA, umol/L	314 (224.65–428.50)	337.9 (256.2–440.9)*†	286.3 (198–395.5)	303.45 (199.5–427.5)	<0.001
Hematological parameters					
WBC, ×10^9^/L	9.09 (6.90–11.90)	8.98 (6.77–11.68)*	9.50 (7.20–12.37)‡	8.78 (6.85–11.82)	0.006
RBC, ×10^12^/L	4.22 (3.71–4.73)	4.26 (3.79–4.73)	4.17 (3.64–4.72)	4.21 (3.68–4.72)	0.034
PLT, ×10^9^/L	303.00 (221.00–388.50)	303.00 (223.00–382.00)	305.00 (216.00–400.00)	300.50 (218.75–385.25)	0.937
Hb, g/dL	116.00 (102.00–130.00)	117.00 (103.00–129.00)*	114.00 (98.00–129.00)	117.00 (100.75–130.00)	0.056
MCV, fL	85.00 (80.00–90.00)	84.00 (80.00–89.00)*†	85.00 (80.00–92.00)	85.00 (81.00–90.00)	<0.001
MCH, pg	28.00 (26.00–30.00)	28.00 (26.00–30.00)	28.00 (26.00–30.00)	28.00 (26.00–30.00)	0.59
MCHC, g/dL	327.00 (318.00–336.00)	328.00 (319.00–337.00)*†	326.00 (314.00–335.00)	327.00 (317.00–336.00)	<0.001

Data are presented as Median (IQR). Symbols indicate a statistically significant difference (*p* < 0.05) in a *post-hoc* pairwise comparison:

*Significant difference between East and West regions.

†Significant difference between East and Central regions.

‡Significant difference between West and Central regions.

BNP, B-type Natriuretic Peptide; NT-proBNP, N-terminal pro-B-type Natriuretic Peptide; CK-MB, Creatine Kinase-MB; cTnI, cardiac Troponin I; ALT, Alanine Aminotransferase; AST, Aspartate Aminotransferase; ALB, Albumin; ALP, Alkaline Phosphatase; Cr, Creatinine; BUN, Blood Urea Nitrogen; UA, Uric Acid; WBC, White Blood Cell Count; RBC, Red Blood Cell Count; PLT, Platelet Count; Hb, Hemoglobin; MCV, Mean Corpuscular Volume; MCH, Mean Corpuscular Hemoglobin; MCHC, Mean Corpuscular Hemoglobin Concentration.

### 3.3 Regional variations in pharmacological management and interventions

Medication utilization patterns reflected regional differences in etiology and clinical practice (Table 4). The use of guideline-directed heart failure therapies, including angiotensin-converting enzyme inhibitors (ACEI: 50.9%) and beta-blockers (BBs: 23.4%), was most prevalent in the Eastern region (*p* < 0.001 vs. West and Central). Antibiotic use was remarkably high in the West (86.1%), significantly exceeding usage in other regions (*p* < 0.001). The utilization of anti-arrhythmic drugs was highest in the East (20.5%, *p* < 0.001), while hormone therapy was more commonly employed in the Central region (53.3%, *p* < 0.001).

**Table 4:** Medication utilization by geographic region.

Variable	Overall, *n* (%)	East, *n* (%)	West, *n* (%)	Central, *n* (%)	*p*-value
ACEI	1,300 (44.8)	699 (50.9)*†	310 (38.7)	291 (40.0)	<0.001
BBs	486 (16.7)	321 (23.4)*†	61 (7.6)‡	104 (14.3)	<0.001
Diuretics	2,504 (86.3)	1,196 (87.0)	702 (87.6)	606 (83.2)	0.022
IA	2,371 (81.7)	1,145 (83.3)*	632 (78.9)	594 (81.6)	0.036
Antibiotics	2008 (69.2)	812 (59.1)*†	690 (86.1)‡	506 (69.5)	<0.001
Anti-arrhythmic drugs	464 (16)	282 (20.5)	89 (11.1)	93 (12.8)	<0.001
Hormones	1,355 (46.7)	613 (44.6)†	354 (44.2)‡	388 (53.3)	<0.001
IVIG	862 (29.7)	435 (31.7)*	193 (24.1)‡	234 (32.1)	<0.001

Data are presented as *n* (%). Symbols indicate a statistically significant difference (p < 0.05) in a post-hoc pairwise comparison:

*Significant difference between East and West regions.

†Significant difference between East and Central regions.

‡Significant difference between West and Central regions.

ACEI, Angiotensin-Converting Enzyme Inhibitors; BBs, Beta-Blockers; IA, Inotropic Agents; IVIG, Intravenous Immunoglobulin. Medication use refers to drugs administered during the index hospitalization.

### 3.4 Clinical outcomes and multivariable regression analyses

Unadjusted outcomes revealed significant regional disparities in mortality (Table 5). The in-hospital mortality rate was lowest in the East (1.7%) and significantly higher in the West (5.6%) and Central (6.3%) regions (*p* < 0.001). The median LOS was comparable across all regions (approximately 12–13 days, *p* = 0.299).

**Table 5:** Outcomes by geographic region.

Variable	Overall	East	West	Central	*p*-value
Death [*n* (%)]	115 (4.0%)	24 (1.7%)*†	45 (5.6%)	46 (6.3%)	<0.001
LOS [median (IQR)]	12 (8–19)	12 (8–19)	13 (8–20)	12 (8–18)	0.299

Symbols indicate a statistically significant difference (*p* < 0.05) in a *post-hoc* pairwise comparison:

*Significant difference between East and West regions.

†Significant difference between East and Central regions.

Although the unadjusted mortality rates were similar between the West and Central regions, multivariable logistic regression, adjusted for age, sex, etiology, disease severity, LVEF, and log_BNP, identified geographic region as a powerful independent predictor of mortality, with a markedly heightened risk associated with the Central region. Compared to the Eastern region, the odds of death were 2.58 times higher in the West and 3.54 times higher in the Central region. Other significant independent predictors of increased mortality included higher ROSS class (Class III-IV vs. I-II) and elevated log_BNP. Notably, the presence of cardiomyopathy was associated with a substantially reduced odds of death (aOR: 0.24; Table 6).

**Table 6:** Logistic regression analysis for predictors of mortality.

Variable	Odds ratio	95% CI	*p*-value
Region (Ref: East)			
West	2.58	1.50, 4.45	0.001**
Central	3.54	2.11, 5.94	<0.001***
Age group (Ref: Newborn)			
Infant	0.48	0.28, 0.81	0.007**
Child	0.44	0.23, 0.85	0.014*
Adolescent	0.53	0.24, 1.18	0.118
Sex (Ref: Female)			
Male	0.74	0.51, 1.1	0.135
CHD (Ref: Absent)			
Present	0.79	0.52, 1.21	0.283
Cardiomyopathy (Ref: Absent)			
Present	0.24	0.13, 0.45	<0.001***
ROSS (Ref: Class I-II)			
Class III-IV	4.12	1.97, 8.59	<0.001***
LVEF (%)	0.985	0.972, 0.998	0.023*
log_BNP	1.15	1.03, 1.29	0.012*

Odds Ratios represent multiplicative effects on mortality risk. OR > 1 indicates higher mortality risk, OR < 1 indicates lower mortality risk. Significance levels: *p < 0.05, **p < 0.01, ***p < 0.001.

A Gamma Generalized Linear Model (GLM) for LOS identified several independent predictors but found no significant independent effect of geographic region after multivariable adjustment. Factors associated with a significantly shorter LOS included older age (Infant, Child, Adolescent vs. Newborn) and the presence of cardiomyopathy (Rate Ratio [RR]: 0.77, *p* < 0.001). Higher ROSS class (RR: 1.12, *p* = 0.001) was associated with longer LOS (Table 7).

**Table 7:** Gamma GLM analysis for predictors of LOS.

Variable	Rate ratio	95% CI	*p*-value
Region (Ref: East)			
West	0.98	0.909, 1.057	0.604
Central	0.939	0.873, 1.01	0.089
Age Group (Ref: Newborn)			
Infant	0.803	0.714, 0.902	<0.001***
Child	0.797	0.702, 0.904	<0.001***
Adolescent	0.795	0.688, 0.919	0.002**
Sex (Ref: Female)			
Male	0.988	0.932, 1.047	0.687
CHD (Ref: Absent)			
Present	1.019	0.952, 1.091	0.593
Cardiomyopathy (Ref: Absent)			
Present	0.774	0.717, 0.835	<0.001***
ROSS (Ref: Class I-II)			
Class III-IV	1.123	1.049, 1.201	0.001**
LVEF (%)	0.994	0.992, 0.996	<0.001***
log_BNP	1.007	0.995, 1.019	0.282

Rate Ratios represent multiplicative effects on length of stay. RR > 1 indicates longer LOS, RR < 1 indicates shorter LOS. Significance levels: *p < 0.05, **p < 0.01, ***p < 0.001.

Sensitivity analyses using categorized LVEF and BNP variables yielded results consistent with the primary models, with geographic region remaining a significant independent predictor of mortality (Supplementary Table S2). The predictors of length of stay were also similar in the categorical analysis (Supplementary Table S3).

## 4 Discussion

This large-scale, national multicenter cohort study provides a stark and quantifiable account of the profound geospatial disparities characterizing PHF care in China. Our analysis revealed that a child’s geographic location—a proxy for a complex web of socioeconomic and healthcare system factors—was powerfully associated with their demographic profile, disease etiology, clinical severity at presentation, therapeutic management, and, most critically, their risk of in-hospital mortality. The findings paint a picture of a tiered healthcare landscape: an Eastern region with a profile resembling developed healthcare systems, a Western region burdened by late-presenting, severe congenital disease in younger infants, and a Central region exhibiting a paradoxical mix of characteristics with the highest adjusted mortality risk.

The most fundamental disparity observed is the stark contrast in the underlying causes of PHF. The Western region was dominated by CHD, particularly complex lesions like VSD and PDA, presenting in significantly younger infants. This pattern strongly suggests systemic delays in the diagnosis and referral of CHD in Western China, likely driven by a combination of factors including limited access to prenatal and postnatal echocardiography, lower healthcare literacy, and financial barriers that prevent families from seeking early care (8, 17, 18). The consequence is that children present later, with more advanced disease, as evidenced by the highest prevalence of ROSS Class III-IV failure, respiratory symptoms, and systemic venous congestion.

Conversely, the Eastern and Central regions had a higher burden of cardiomyopathy, a finding more aligned with PHF etiologies in high-income countries (19, 20). This shift suggests better early diagnosis and intervention for CHD in these regions, allowing other chronic myocardial diseases to constitute a larger proportion of advanced heart failure cases. The older age at presentation in the East further supports this, indicating a system where immediately life-threatening congenital defects are identified and corrected earlier, leaving a cohort of patients with progressive, chronic conditions.

The echocardiographic and biomarker data provide a pathophysiological underpinning to these clinical observations. The preserved LVEF and LVFS in Western patients, despite their severe clinical scores, are consistent with a predominantly volume-overloaded state from large left-to-right shunts (e.g., VSD, PDA) (3, 21). Their significantly elevated pulmonary artery pressure underscores the consequences of delayed intervention, leading to accelerated pulmonary vascular remodeling (22–24). In contrast, the Eastern cohort’s markedly lower LVEF aligns with the high prevalence of dilated cardiomyopathy, reflecting a primary systolic dysfunction phenotype (25, 26).

The biomarker profile further highlights regional differences in myocardial stress and injury. The Central region had the highest median BNP and CK-MB levels, indicating profound myocardial strain and injury, potentially exacerbated by a high burden of severe infection. Interestingly, the West had the lowest BNP levels, which, in the context of their severe clinical status, is a noteworthy finding. This apparent discrepancy can be explained by the unique pathophysiology of large, unrepaired left-to-right shunts that dominated this cohort. In chronic volume-overload states, the right ventricle and atria bear a significant portion of the hemodynamic burden, and BNP secretion may not rise in a linear fashion compared to pressure-overload or primary systolic dysfunction states (27–29). Furthermore, BNP interpretation in infancy is inherently complex. Baseline BNP levels are highly age-dependent, being physiologically higher in the first weeks of life and then declining (30, 31). Furthermore, the relative elevation of BNP is more significant than the absolute value. Therefore, the lower absolute BNP in the Western cohort, while the lowest in our study, may still represent a substantial elevation from the normal range for infants of a similar age (32). Therefore, the lower absolute BNP in the Western cohort likely reflects a combination of a different hemodynamic profile and the younger age of the patients, rather than a lesser degree of clinical severity. This pathophysiological variation challenges the use of BNP as a uniform severity adjustor in our model, potentially influencing the estimated geographic risk. The elevated liver enzymes (ALT, AST) in the West and Central regions are classic signs of systemic venous congestion and end-organ hypoperfusion, further corroborating the finding of more advanced, decompensated heart failure at presentation (33).

The pharmacological management patterns reveal a concerning gap in the application of GDMT, a challenge well-documented in pediatric heart failure due to a weaker evidence base and significant practice variation (34). The use of ACEIs and BBs was significantly lower in the West and Central regions compared to the East. This disparity may be attributed to several factors documented in the pediatric literature: a lack of specialized pediatric cardiology expertise and dedicated heart failure programs, which are unevenly distributed (35); justifiable concerns about extrapolating adult guidelines to infants with complex shunt physiology, where safety and efficacy are less certain (3, 36); or a systemic focus on acute surgical stabilization that can sometimes come at the expense of longitudinal medical optimization in the outpatient setting (37).

The extraordinarily high rate of antibiotic use in the West is particularly striking. This likely reflects a higher suspicion or prevalence of concurrent respiratory infections in a population with chronic pulmonary over-circulation, or potentially its use as a prophylactic measure in malnourished, vulnerable infants (38–40). The high utilization of hormones in the Central region is more difficult to interpret but may be linked to a higher incidence of myocarditis or a practice pattern favoring immunomodulation in severe, inflammatory presentations (41, 42). The concentrated use of advanced interventions like pacemakers and radiofrequency ablation almost exclusively in the East points to a critical centralization of complex electrophysiological care, creating a “postcode lottery” for children with arrhythmic complications of heart failure. The most critical finding of this study is that geographic region is an independent, powerful predictor of in-hospital mortality. Even after rigorous adjustment for age, etiology, disease severity (ROSS class, LVEF), and neurohormonal activation (BNP), children in the West faced a 2.58-fold higher odds of death, while those in the Central region faced a staggering 3.54-fold higher odds, compared to their counterparts in the East.

The elevated risk in the West can be reasonably attributed to the “double burden” of later presentation with more severe, advanced-stage disease and potentially less access to timely, specialized surgical intervention. The situation in the Central region is more complex and alarming. This is underscored by the finding that, despite a patient profile that was, in some aspects, intermediate between the East and West (e.g., age, CHD prevalence), it exhibited the highest adjusted mortality. This suggests that factors beyond what we measured—such as quality of intensive care, surgical outcomes, post-operative management, or unmeasured socioeconomic and nutritional factors—may be driving this excess mortality (43–45). The exceptionally high burden of severe infection, coupled with biomarker evidence of severe myocardial injury, points to a high-risk phenotype of ‘heart failure with multimorbidity,’ where systemic infection may trigger catastrophic decompensation. Unmeasured socioeconomic and nutritional factors likely contribute, but the data strongly suggest that the confluence of advanced heart failure and severe infection is a critical determinant of the heightened mortality observed in the Central region.

The protective association of cardiomyopathy with mortality (aOR: 0.24) is counterintuitive but may reflect the more chronic, manageable nature of many cardiomyopathies compared to critically ill, late-presenting infants with complex CHD, who dominated the high-mortality cohorts. This finding likely illustrates a disparity in acute risk profiles rather than an inherent biological advantage of cardiomyopathy, with the elevated mortality driven substantially by the high perioperative risk of late-presenting complex CHD. It may also indicate that centers with expertise in cardiomyopathy management (more prevalent in the East) achieve excellent outcomes, pulling down the overall risk for this group.

Several limitations inherent to this study’s design should be considered when interpreting its findings. First, the retrospective design inherently limits the availability of certain variables and is susceptible to unmeasured confounding, despite adjustments for key clinical factors in our analysis. Second, our cohort was drawn exclusively from major referral hospitals. While this reflects the centralized model for specialized pediatric cardiac care in China, it introduces referral bias by excluding children who died before referral or were managed locally. Importantly, children in remote and less developed regions face significant barriers to accessing tertiary centers and optimal care (12–14, 46, 47). Therefore, this bias likely leads to an underestimation of both the true mortality and the scale of the observed disparities, as both severely ill patients who die before referral and less severe cases treated locally are excluded from our tertiary-center cohort. Third, we lacked granular data on socioeconomic status, parental education, precise travel distances, and specific details on surgical timing and outcomes, all of which are critical mediators of the disparities we observed. Consequently, while our analysis confirms that geographic region is a strong independent predictor of mortality after accounting for clinical factors, it cannot distinguish the extent to which this effect is mediated by pre-hospital barriers (e.g., income, distance, education) versus differences in hospital-level care quality. The variable ‘region’ encapsulates all these unmeasured systemic determinants. Fourth, the use of in-hospital mortality as the primary outcome does not capture post-discharge deaths, which may be substantial, particularly in regions with poor follow-up care. Fifth, the observed regional variations should be interpreted with the understanding that some heterogeneity exists within each region, an inherent feature of real-world, national cohort data that enhances the generalizability of our findings. Furthermore, the exclusion of individual records with >20% missing data may introduce selection bias. A meaningful comparison of characteristics (e.g., outcomes, region) between excluded and included patients was not feasible, as the defining feature of the excluded group was the critical absence of this data. These limitations directly inform critical avenues for future research. Prospective, population-based studies that include primary and secondary healthcare centers are essential to capture the true burden of disease and avoid referral bias. Furthermore, subsequent investigations must integrate detailed socioeconomic metrics, travel data, and treatment process measures to fully elucidate the mechanisms behind the observed disparities. Finally, establishing national registries with long-term follow-up is paramount to understanding the full impact of PHF beyond the initial hospitalization and to evaluating the effectiveness of future interventions aimed at achieving healthcare equity.

## 5 Conclusion

In conclusion, this study provides robust evidence that a child’s geographic location in China is a powerful independent predictor of survival and care quality for pediatric heart failure. The profound disparities in patient characteristics, management, and outcomes between the East, West, and Central regions are not merely variations in practice but reflect deep-seated inequities in the healthcare ecosystem. Addressing these disparities requires moving beyond a one-size-fits-all approach and implementing targeted, region-specific strategies to build capacity, improve access, and standardize care. Ensuring that every child with heart failure in China, regardless of their birthplace, has an equal chance at survival is an urgent moral and public health imperative.

## Data Availability

The raw data supporting the conclusions of this article will be made available by the authors, without undue reservation.
