# Supplementary material for: Geospatial disparities in pediatric heart failure care in China: a multicenter cohort study
Source: Front Public Health. 2026 Jan 16;13:1737404. doi: 10.3389/fpubh.2025.1737404 (PMC12857641; doi:10.3389/fpubh.2025.1737404)
Supplement: Supplementary file 1 [file Table_1.DOCX]

**Supplementary Materials**

|  | **Table S1 : List of All Hospitals by Region** |
| --- | --- |
| **Hospitals in East Region** | |
| 1 | Children's Hospital Affiliated to Fudan University |
| 2 | Children's Hospital Affiliated to Zhejiang University |
| 3 | Guangzhou Women and Children's Center |
| 4 | Shanghai Children's Medical Center |
| 5 | Shengjing Hospital Affiliated to China Medical University |
| 6 | Xinhua Hospital |
| 7 | Beijing Anzhen Hospital |
| 8 | Fuwai Hospital |
| 9 | Guangdong Provincial People's Hospital |
| 10 | Hebei Provincial Children's Hospital |
| 11 | Jinan Children's Hospital |
| 12 | Nanjing Children's Hospital |
| 13 | Qilu Hospital of Shandong University |
| 14 | Qingdao Women and Children's Hospital |
| 15 | The Second Hospital of Hebei Medical University |
| 16 | Tianjin Children's Hospital |
| **Hospitals in West Region** | |
| 1 | Children's Hospital Affiliated to Chongqing Medical University |
| 2 | PICU of the Affiliated Hospital of Southwest Medical University |
| 3 | Sichuan Provincial People's Hospital |
| 4 | Xi'an Children's Hospital |
| 5 | Tibet Autonomous Region People's Hospital |
| 6 | The First Affiliated Hospital of Shihezi University Medical College |
| 7 | Sichuan Provincial Maternal and Child Health Hospital |
| **Hospitals in Central Region** | |
| 1 | Department of Pediatrics, Anhui Provincial Hospital |
| 2 | Henan Provincial Children's Hospital |
| 3 | Hunan Children's Hospital |
| 4 | Inner Mongolia People's Hospital |
| 5 | Jiangxi Provincial Children's Hospital |
| 6 | Jilin University First Hospital |
| 7 | The First Affiliated Hospital of Zhengzhou University |

| **Table S2. Logistic Regression Analysis for Predictors of Mortality**  **(with LVEF and BNP Categories)** | | | |
| --- | --- | --- | --- |
| **Variable** | **Odds Ratio** | **95% CI** | **p-value** |
| Region (Ref: East) | | | |
| West | 2.50 | 1.45, 4.30 | 0.001** |
| Central | 3.71 | 2.21, 6.23 | <0.001*** |
| Age Group (Ref: Newborn) | | | |
| Infant | 0.46 | 0.27, 0.79 | 0.005** |
| Child | 0.43 | 0.23, 0.82 | 0.011* |
| Adolescent | 0.52 | 0.24, 1.15 | 0.107 |
| Sex (Ref: Female) | | | |
| Male | 0.74 | 0.50, 1.08 | 0.120 |
| CHD (Ref: Absent) | | | |
| Present | 0.79 | 0.52, 1.20 | 0.271 |
| Cardiomyopathy (Ref: Absent) | | | |
| Present | 0.28 | 0.15, 0.52 | <0.001*** |
| ROSS (Ref: Class I-II) | | | |
| Class III-IV | 4.55 | 2.18, 9.52 | <0.001*** |
| LVEF (%) (Ref: ≤40%) | | | |
| 41–49% | 1.80 | 0.95, 3.41 | 0.072 |
| ≥50% | 0.71 | 0.41, 1.22 | 0.214 |
| **BNP (Ref: <100 pg/mL)** |  |  |  |
| 100–400 | 0.94 | 0.35, 2.48 | 0.897 |
| >400 | 1.76 | 0.91, 3.39 | 0.091 |
| Odds Ratios represent multiplicative effects on mortality risk. OR > 1 indicates higher mortality risk, OR < 1 indicates lower mortality risk. Significance levels: *p < .05, **p < .01, ***p < .001. | | | |

| **Tabe S3. Gamma GLM Analysis for Predictors of Length of Stay (LOS)**  **(with LVEF and BNP Categories)** | | | |
| --- | --- | --- | --- |
| **Variable** | **Rate Ratio** | **95% CI** | **p-value** |
| Region (Ref: East) | | | |
| West | 0.97 | (0.90, 1.05) | 0.447 |
| Central | 0.93 | (0.87, 1.00) | 0.064 |
| Age Group (Ref: Newborn) | | | |
| Infant | 0.79 | (0.71, 0.89) | <0.001*** |
| Child | 0.79 | (0.70, 0.90) | <0.001*** |
| Adolescent | 0.80 | (0.69, 0.92) | 0.002** |
| Sex (Ref: Female) | | | |
| Male | 0.99 | (0.93, 1.05) | 0.729 |
| CHD (Ref: Absent) | | | |
| Present | 1.02 | (0.95, 1.09) | 0.619 |
| Cardiomyopathy (Ref: Absent) | | | |
| Present | 0.78 | (0.72, 0.84) | <0.001*** |
| ROSS (Ref: Class I-II) | | | |
| Class III-IV | 1.12 | (1.04, 1.20) | 0.001** |
| **LVEF (%) (Ref: ≤40%)** | | | |
| 41–49% | 0.84 | (0.76, 0.94) | 0.001** |
| ≥50% | 0.80 | (0.74, 0.87) | <0.001*** |
| **BNP (Ref: <100 pg/mL)** | | | |
| 100–400 | 1.01 | (0.90, 1.13) | 0.842 |
| >400 | 1.02 | (0.94, 1.11) | 0.595 |
| Rate Ratios represent multiplicative effects on length of stay. RR > 1 indicates longer LOS, RR < 1 indicates shorter LOS. Significance levels: *p < .05, **p < .01, ***p < .001. | | | |

# Table S4. List of researchers (Arranged from high to low according to their contribution)

| **S.No.** | **Researchers along with their Affiliations** |
| --- | --- |
| 1 | Children’s Hospital of Chongqing Medical University (Zhilin Huang, Huichao Sun, Lingjuan Liu, Tiewei Lv); |
| 2 | Henan Children's Hospital, Children's Hospital Afliated to Zhengzhou University (Fangjie Wang, Xiaochen Yan, Xiaoli Yao, Yingying Li); |
| 3 | Hunan Children's Hospital (Zhi Chen); |
| 4 | Shanghai Children’s Medical Center, School of Medicine, Shanghai Jiao Tong University (Ying Guo); |
| 5 | The Affiliated Hospital of Southwest Medical University (Xing Shen); |
| 6 | Children's Hospital of Fudan University (Fang Liu, Xuecun Liang, Yixiang Lin, Lan Ye); |
| 7 | Inner Mongolia People's Hospital (Hua Zhu, Yanyan Liang); |
| 8 | Shengjing Hospital of China Medical University (Yanlin Xing, Hong Wang, Wei Liu); |
| 9 | Fuwai hospital，Chinese Academy of Medical Sciences, Peking Union Medical College (Huili Zhang); |
| 10 | Hebei Children's Hospital (Yingqian Zhang, Bo Li); |
| 11 | Children's Hospital, Zhejiang University School of Medicine (Chunhong Xie, Yue Huang, Jianmei Zhu, Zhe Lin); |
| 12 | Children's Hospital of Nanjing Medical University (Shiwei Yang, Lianfu Ji); |
| 13 | Tianjin Children's Hospital (Shuhua Xing, Jiegang Deng); |
| 14 | The First Bethune Hospital of Jilin University (Yanyan Han, Shu Nie, Huaqing Sun); |
| 15 | The Second Hospital of Hebei Medical University (Lin Feng, Wei Xu, Haoyun Zhao); |
| 16 | The First Affiliated Hospital of Zhengzhou University (Jindou An, Song Feng); |
| 17 | Guangzhou Women and Children's Medical Center，Guangzhou Medical University (Li Zhang, Xiaofei Xie); |
| 18 | Qilu Hospital of Shandong University (Cuifen Zhao, Minmin Wang, Haizhao Zhao); |
| 19 | Qingdao Women and Children's Hospital (Zipu Li, Benzhen Wang, Guangsong Shan); |
| 20 | Xi'an Children's Hospital (Juanli Wang, Hongyu Xiao, Huan Li); |
| 21 | Tibet Autonomous Region People's Hospital (Bianbazhuoga, Mei Chen, Longya Qiao, Xiangyan Zhong, Yude Ma); |
| 22 | Jiangxi Provincial Children's Hospital (Junkai Duan, Fei Xu, Yunguo Zhou, Fang Xu); |
| 23 | Sichuan Academy of Medical Sciences & Sichuan Provincial People's Hospital (Qian Peng, Xiaoping Hu, Bo Li); |
| 24 | Guangdong Provincial People's Hospital, Guangdong Academy of Medical Sciences (Zhaofeng Xie, Yan Guan, Zhiwei Zhang, Shuishu Wang); |
| 25 | Anhui Provincial Hospital (Mei Xiong); |
| 26 | Xinhua Hospital Affiliated to Shanghai Jiaotong University School of Medicine (Yurong Wu); |
| 27 | Children's Hospital Affiliated to Shandong University, Jinan Children's Hospital (Lei Li, Xinxiang Li); |
| 28 | Beijing An Zhen Hospital of the Capital University of Medical Sciences (Yongmei Liang); |
| 29 | The First Affiliated Hospital of Shihezi University (Jinyong Pan, Qiang Gu, Fang Jiang); |
| 30 | Sichuan Provincial Maternity and Child Health Care Hospital (Xianmin Wang). |
